# Prediction of Cancer Proteins by Integrating Protein Interaction, Domain Frequency, and Domain Interaction Data Using Machine Learning Algorithms

**DOI:** 10.1155/2015/312047

**Published:** 2015-03-17

**Authors:** Chien-Hung Huang, Huai-Shun Peng, Ka-Lok Ng

**Affiliations:** ^1^Department of Computer Science and Information Engineering, National Formosa University, 64 Wen-Hwa Road, Huwei, Yunlin 63205, Taiwan; ^2^Department of Biomedical Informatics, Asia University, Wufeng Shiang, Taichung 41354, Taiwan; ^3^Department of Medical Research, China Medical University Hospital, China Medical University, Taichung 40402, Taiwan

## Abstract

Many proteins are known to be associated with cancer diseases. It is quite often that their precise functional role in disease pathogenesis remains unclear. A strategy to gain a better understanding of the function of these proteins is to make use of a combination of different aspects of proteomics data types. In this study, we extended Aragues's method by employing the protein-protein interaction (PPI) data, domain-domain interaction (DDI) data, weighted domain frequency score (DFS), and cancer linker degree (CLD) data to predict cancer proteins. Performances were benchmarked based on three kinds of experiments as follows: (I) using individual algorithm, (II) combining algorithms, and (III) combining the same classification types of algorithms. When compared with Aragues's method, our proposed methods, that is, machine learning algorithm and voting with the majority, are significantly superior in all seven performance measures. We demonstrated the accuracy of the proposed method on two independent datasets. The best algorithm can achieve a hit ratio of 89.4% and 72.8% for lung cancer dataset and lung cancer microarray study, respectively. It is anticipated that the current research could help understand disease mechanisms and diagnosis.

## 1. Introduction

It has been known for a long time that cancer is a result of loss of cell cycle control. The loss of control is a result of series of genetic mutations involving activation of proto-oncogenes to oncogenes and inactivation of tumor-suppressing genes. Oncogenes and tumor suppressors may cause cancer by alternating the transcription factors, such as the p53 and ras oncoproteins, which in turn control expression of other genes. Therefore, understanding how oncoprotein-oncoprotein interacts and how oncoproteins drive the cell division cycle is indispensable for the study of molecular oncology. Predicting novel cancer-related proteins is an important topic in biomedical research; experimental techniques such as microarrays are being used to characterize cancer. However, the process could be time consuming and labor-intensive. Nagaraj and Reverter [1] proposed a Boolean logic based approach to predict colorectal cancer genes. Li et al. [2] took GO enrichment scores and KEGG enrichment scores as features to predict retinoblastoma related genes. The above two studies are confined to predict specific cancers. For general types of cancers, Hosur et al. [3] combined linear programming formulation for interface alignment to predict cancer related PPIs. Aragues et al. [4] used PPI data to predict cancer-related proteins. In this study, we extended Aragues's study by employing PPI data and domain information to attain improved performance.

Protein-protein interactions are inherent in almost every cellular process. In fact, PPI is the core of the entire interactomics system in living cells. PPI appears when two or more proteins bind together and perform a biological function [5]. Almost all major research topics in molecular biology involve PPI such as cellular function [6], genetic diseases [7], conserved patterns [8], and homologous relationships [9]. The recent availability of PPI data has made it possible to study human disease at a system level. It is reported that since disease genes exhibit an increased tendency for their protein products to interact with one another, they tend to be coexpressed in specific tissues and display coherent functions [10]. Ideker and Sharan reported in a review article [11] on the applications of PPI networks to study disease in four major areas: (i) identifying new disease genes, (ii) studying their network properties; (iii) identifying disease-related subnetworks, and (iv) performing network-based disease classification. Another study [12] investigated the human cancer PPI network from a structural perspective, that is, protein interactions through their interfaces. Their findings indicated that cancer-related proteins have smaller, more planar, more charged, and less hydrophobic binding sites than noncancer proteins.

It is known that proteins are composed of multiple functional domains. A domain is a unit of function associated with different catalytic functions or binding sites, as found in enzymes or regulatory proteins. It is hypothesized that cancer proteins, also known as tumor associated genes, may share common functional domains [13], and thus a weighted domain score for each tumor associated gene's domain is determined. Novel cancer proteins are determined by translating full cDNA sequences to the corresponding protein sequences and calculating the weighted domain scores. Another work [14] used established methods to identify the network topology of a cancer protein network. They showed that cancer proteins contain a high ratio of structural domains, which have a high propensity for mediating protein interactions. Recently, Clancy et al. designed a statistical method to infer the physical interactions between two complexes for the human and yeast species [15]. Domains such as the immunoglobulin domain, Zinc-finger, and the protein kinase domains are the top three most frequently observed cancer protein domains. Many other works also employed PPI and DDI to characterize disease networks [7, 16–20]. In our previous works [21, 22], a one-to-one DDI model was proposed to obtain specific sets of DDI for oncoproteins and tumor suppressor proteins, respectively. Three specific sets of DDI, that is, oncoprotein and oncoprotein, tumor suppressor protein and tumor suppressor protein, and oncoprotein and tumor suppressor protein, are derived from their PPIs.

Weka (http://www.cs.waikato.ac.nz/~ml/weka/) [23] is a well known software tool which provides environments related to machine learning, data mining, text mining, predictive analysis, and business analysis. Machine learning and data mining algorithms have been widely used in bioinformatics and computational biology [24–28]. The present authors adopted amino acid composition profile information with the SVM classifier to improve protein complexes classification [29]. Additionally, we also proposed identifying microRNAs target of* Arabidopsis thaliana* by integrating prediction scores from PITA, miRanda, and RNAHybrid algorithms [30]. Recently, Li et al. [31] used random forest machine learning algorithm and topology features to identify the functions of protein complexes.

In this research, we began by collecting cancerous protein interaction data. That is, only interactions involved with cancer proteins are considered. These types of interactions are known as cancerous PPIs. Noncancerous protein interaction means either one or both of the proteins are not yet identified in relation to cancer. Given the cancerous PPIs, a set of DDI rules for cancer proteins are derived. In addition to this set of DDI, we also considered other features: the weighted domain frequency scores (DFS): DFS_C for cancer proteins and DFS_X for noncancer proteins and the cancer linker degree (CLD) score. A total of four features (DDI, DFS_C, DFS_X, and CLD) are adopted to make novel cancer protein predictions by using 39 machine learning algorithms from the Weka tool. In addition, we also verified the accuracy of the predictive model on two independent datasets. Finally, using differentially expressed genes found in lung cancer microarray data as a case study, we discovered some potential cancer genes for further experimental investigation.

## 2. Methods

### 2.1. System Flowchart

**Figure 1:**
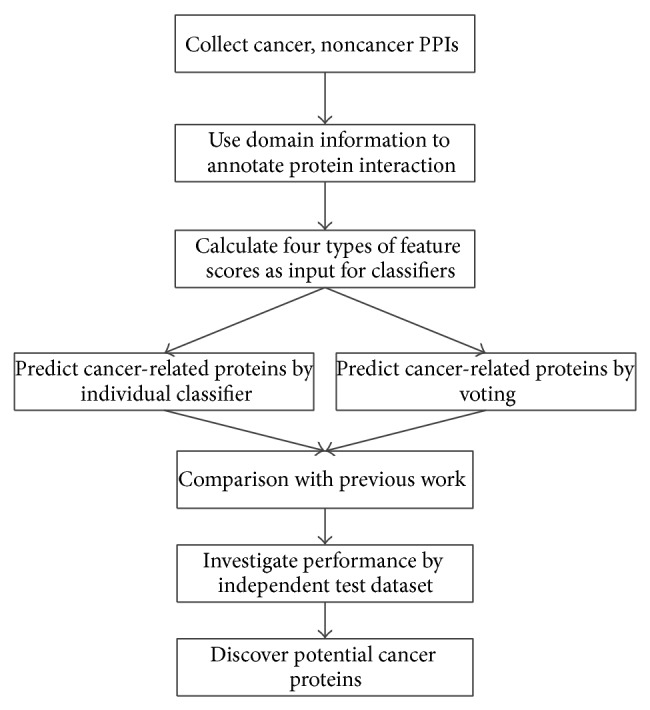
System flowchart for this study.

In this study, a system was set up to predict cancer proteins by integrating four types of features. Firstly, cancer and noncancer PPIs were collected from biological databases, and then those interactions were annotated by using the domain information. Next, we determined four feature scores; data normalization is needed to ensure consistency in their distribution. The system flowchart of this study is illustrated in Figure 1.

### 2.2. Data Sources and Datasets Generation

Cancer proteins (tumor suppressor protein (TSP) or oncoprotein (OCP)) of the learning dataset were integrated from Institute of Biopharmaceutical Sciences of Taiwan National Yang Ming University, Tumor Associated Gene (TAG, http://www.binfo.ncku.edu.tw/TAG/GeneDoc.php) database [13], and Memorial Sloan-Kettering Cancer Center (MSKCC, http://cbio.mskcc.org/research/cancergenomics/index.html). Noncancer proteins were from BioGrid (http://thebiogrid.org/) [32]. On the other hand, cancer proteins of the independent test dataset for Case Study 1 were obtained from two resources, that is, Online Mendelian Inheritance in Man (OMIM, http://www.ncbi.nlm.nih.gov/omim) and the human lung cancer database (HLungDB) [33].

Protein domain information was downloaded from PFam database (http://pfam.xfam.org/) [34]. PPIs for both cancer and noncancer proteins were retrieved from BioGrid database. The Swiss ID of proteins was obtained from Swiss-Prot Database (http://www.expasy.org/sprot/).

**Figure 2:**
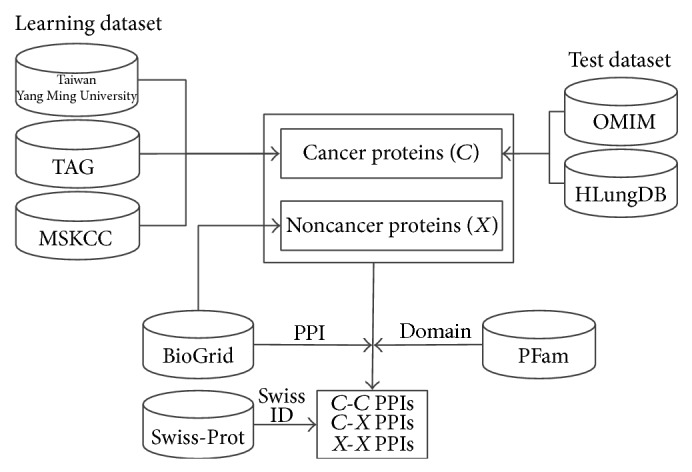
Data sources and datasets generation.

There are three classified combinations of protein interaction adopted as input data in the current study: “*C*-*C*” indicates cancer-cancer protein interaction, “*C*-*X*” indicates cancer-noncancer protein interaction, and “*X*-*X*” indicates noncancer-noncancer protein interaction.  Figure 2 depicts input data sources and dataset generation of this research.

### 2.3. Feature Scores Generation

We derived four feature scores based on three different approaches, which are domain-domain interaction, weighted domain frequency, and cancer linker degree. For the domain-domain interactions, it may happen that some relationships are derived from homologous sequences, which may produce a bias in the 10-fold validation; therefore, some redundancies (by a certain homology degree that includes domains) should be removed. By extracting the homologous PPIs by using the “UniRef50” dataset obtained from the UniProt Reference Clusters (UniRef, http://www.uniprot.org/uniref/), we found that, among the 123751 edges derived from BioGrid database, only 431 edges (approximately 0.348%) are homologous PPIs and 86 edges are cancer PPIs; it means that most of the PPIs are not homologous PPIs. We removed the 431 PPIs and the remaining 123320 PPIs are used in our experiment. In addition, the 431 homologous PPIs comprise 180 proteins, in which 179 proteins still appear in other nonhomologous PPIs; therefore, the total number of domains, that is, 3970, remains unchanged.

### 2.4. One-to-One Domain Interaction Model

Assuming that proteins *P*_*i*_ and *P*_*j*_ contain *M* and *N* domains, respectively, and then given an interacting protein pair (*P*_*i*_, *P*_*j*_), one considers that there are *MN* possible domain pairs. The set of domain pairs of two proteins *P*_*i*_ and *P*_*j*_, *S*_*i*,*j*_, is defined by

(1) $$\begin{array}{c} S_{i,j}=\left\{S\left(P_{i}\right)\times S\left(P_{j}\right)\right\}, \end{array}$$

where *S*(*P*_*i*_) and *S*(*P*_*j*_) denote sets of protein domains in proteins *P*_*i*_ and *P*_*j*_, respectively, and × denotes the Cartesian product of two sets *S*(*P*_*i*_) and *S*(*P*_*j*_).

To measure the likelihood of a DDI combination, a DDI pair interaction matrix *I* is introduced. The element *I*_*α*,*β*_ denotes the weighted combination probability of a domain pair (*α*, *β*) for a given protein pair (*P*_*i*_, *P*_*j*_), and it is given by

(2) Iα,β =∑Pi,Pj1SPi∗SPj if  α  in  SPi  and  β  in  SPj,

where |*S*(*P*_*i*_)| and |*S*(*P*_*j*_)| denote the set sizes of *S*(*P*_*i*_) and *S*(*P*_*j*_), respectively, and ∗ is the multiplication operation; the summation is over all possible protein pairs of (*P*_*i*_, *P*_*j*_) such that *α* and *β* are an element of *S*(*P*_*i*_) and *S*(*P*_*j*_), respectively. Subsequently, protein domains are randomized while maintaining the number of domain assignments for each protein the same as the original set. The randomized counterpart of *I*_*α*,*β*_, 〈*I*_*α*,*β*_^rand^〉, is performed in order to justify the protein domain pair calculation. Then, the domain pair score of the domain pair (*α*, *β*), *R*_*α*,*β*_, is defined by

(3) $$\begin{array}{c} R_{\alpha ,\beta }=\frac{I_{\alpha ,\beta }}{\langle I_{\alpha ,\beta }^{\text{rand}}\rangle }, \end{array}$$

where 〈*I*_*α*,*β*_^rand^〉 denotes the ensemble average (we randomized the data 20 and 40 times, and it was found that the results converge after 40 times) of the randomized counterpart of *I*_*α*,*β*_. This result provides a criterion to rank the domain pairs. If the ratio *R*_*α*,*β*_ is larger than one, then the correlation is stronger than the randomized counterpart, so the domain pair (*α*, *β*) is a preferred DDI relation.

Given the set of domain annotation for any two proteins, one can turn around and compute a score that signifies PPI based on the set of *R*_*α*,*β*_ values for DDI. This derived PPI score can answer the question whether any two proteins interact or not given their domain components. The DDI score for the protein pair (*P*_*i*_, *P*_*j*_), DDI_*i*,*j*_, is defined as follows:

(4) $$\begin{array}{c} \text{DD}{\text{I}}_{i,j}=\sum_{\alpha \in S(P_{i}),\beta \in S(P_{j})}R_{\alpha ,\beta }\;\text{if}\;\;R_{\alpha ,\beta }>1, \end{array}$$

where *S*(*P*_*i*_) and *S*(*P*_*j*_) denote the set of domains in proteins *P*_*i*_ and *P*_*j*_, respectively.

### 2.5. Weighted Domain Frequency Score (DFS)

The two feature scores (DFS_C and DFS_X) are defined in this section as the variations from the study by Chan [35]. Among the total of 3970 collected human domain types, the numbers of 381 and 2750 of them appear only in cancer proteins and noncancer proteins, respectively, and 839 of them appear in both cancer proteins and noncancer proteins. This result supports the propensity that certain domain types reside in cancer and noncancer proteins.

Let *C* = (*C*_1_, *C*_2_,…, *C*_*s*_) represent the set of cancer proteins, and let *D* ^*C*^ = (*d*_1_^*C*^, *d*_2_^*C*^,…, *d*_*m*_^*C*^) be the set of domain types that appear in the cancer proteins; similarly, let *X* = (*X*_1_, *X*_2_,…, *X*_*t*_) denote the set of noncancer proteins, and let *D* ^*X*^ = (*d*_1_^*X*^, *d*_2_^*X*^,…, *d*_*n*_^*X*^) be the set of domain types that appear in noncancer proteins. For each domain *α*, let *C*(*α*) and *X*(*α*) denote the numbers of occurrence of domain *α* in cancer proteins and noncancer proteins, respectively. A higher score value suggested that the domain has a high propensity which resides in cancer or noncancer proteins. Then, two weighted DFS values for the protein pair (*P*_*i*_, *P*_*j*_), DFS_*C*_*i*,*j*_ and DFS_*X*_*i*,*j*_, are defined by the following, respectively:

(5) $$\begin{array}{c} \text{DFS}\_C_{i,j}=\frac{\sum_{\alpha \in S\left(P_{i}\right)}C\left(\alpha \right)+\sum_{\beta \in S\left(P_{j}\right)}C\left(\beta \right)}{m+n}, \end{array}$$

(6) $$\begin{array}{c} \text{DFS}\_X_{i,j}=\frac{\sum_{\alpha \in S\left(P_{i}\right)}X\left(\alpha \right)+\sum_{\beta \in S\left(P_{j}\right)}X\left(\beta \right)}{m+n}, \end{array}$$

where *m* and *n* are the total number of domain types that appear in cancer and noncancer proteins, respectively, and *S*(*P*_*i*_) and *S*(*P*_*j*_) denote sets of protein domains in proteins *P*_*i*_ and *P*_*j*_, respectively.

The weighted DFS is adopted to measure the propensity of domain occurrence in cancer and noncancer proteins.

### 2.6. Cancer Linker Degree (CLD)

The last feature is named the cancer linker degree (CLD) score which was adopted from the model proposed by Aragues et al. [4]. In organisms, proteins interact with each other to form a protein complex in order to perform special functions. We can conjecture the category of function and the level of activity by observing their interaction partners. For a given protein pair (*P*_*i*_, *P*_*j*_), let *n* ^*C*^(*P*_*i*_, *P*_*j*_) and *n* ^*X*^(*P*_*i*_, *P*_*j*_) denote the number of adjacent cancer proteins and noncancer proteins in PPI, respectively. Then, the cancer linker degree score for the protein pair (*P*_*i*_, *P*_*j*_), CLD_*i*,*j*_, is defined by

(7) $$\begin{array}{c} \text{CL}{\text{D}}_{i,j}=\frac{n^{C}\left(P_{i},P_{j}\right)}{n^{C}\left(P_{i},P_{j}\right)+n^{X}\left(P_{i},P_{j}\right)}. \end{array}$$

**Figure 3:**
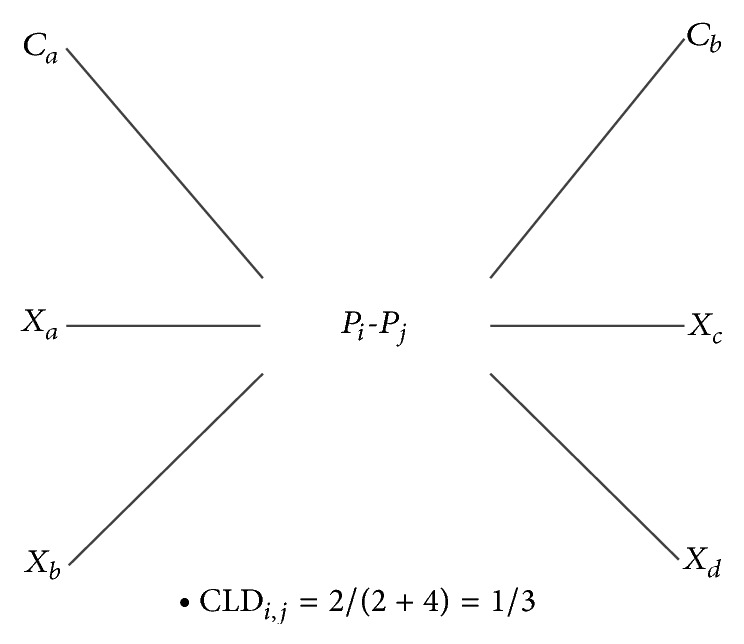
The calculation of score CLD for the protein pair (*P*_*i*_, *P*_*j*_).

As an illustration, an example is presented in Figure 3, where *C*_*a*_ and *C*_*b*_ are cancer proteins, and *X*_*a*_, *X*_*b*_, *X*_*c*_, and *X*_*d*_ are noncancer proteins.

The CLD score represents the interaction ratio for a specified PPI interacting with a cancer partner. If the CLD score is close to one, it implies that the interaction edge is connecting many cancer nodes and could be located in the core of the cancer-related protein clusters.

### 2.7. Data Normalization

The four features scores consist of DDI score (DDI_*i*,*j*_), weighted domain frequency scores (DFS_*C*_*i*,*j*_ and DFS_*X*_*i*,*j*_), and cancer linker degree score (CLD_*i*,*j*_). Due to the fact that the distributions of the numerical values for the above four features are not consistent between each other, data normalization is needed. For the value after normalization, *Y* is defined by

(8) $$\begin{array}{c} Y=\frac{X^{D,W,C}-\min}{\max-\min}, \end{array}$$

where *X* ^*D*,*W*,*C*^ denotes the unnormalized feature value and max and min are the maximum and minimum values for *X* ^*D*,*W*,*C*^, respectively.

### 2.8. Machine Learning Algorithms and Performance Statistical Measures

Since different choices of machine learning algorithms resulted in different predictions of performance, we conducted several comprehensive experiments to determine the optimal combinations of the algorithms. Thirty-nine machine learning algorithms in Weka are discussed in this study. Readers may refer to [23] for detailed descriptions about these algorithms. According to Weka, machine learning algorithms are divided into six classification types, that is, “Bayes” (6 algorithms), “functions” (6 algorithms), “Misc” (2 algorithms), “lazy” (4 algorithms), “rules” (9 algorithms), and “trees” (12 algorithms). A rigorous 10-fold cross validation test is performed to test the classification performance.

Six statistical measures are introduced to quantify the prediction performance, that is, accuracy (ACC), specificity (SPE), sensitivity (SEN), *F*-score (*F*1), Matthew's correlation coefficient (MCC), and positive predictive value (PPV), which are defined in terms of TP, TN, FP, and FN, where they denote true positive, true negative, false positive, and false negative events, respectively. Their definitions are listed in (9). SPE and SEN measure how well a true cancer protein or a true noncancer protein is identified. *F*1 conveys the balance between SPE and SEN. ACC and MCC provide an integrative measure of correct identification. PPV is positive predictive fraction. In addition, the AUC (area under the curve) score, which provides a global performance evaluation, is also included:

(9) ACC=TP+TNTP+TN+FP+FN,SPE=TNTN+FP,SEN=TPTP+FN,F1=2×SPE×SENSPE+SEN×100%,MCC=TP∗TN−FN∗FPTP+FN∗TN+TP∗TP+FP∗TN+FN,PPV=TPTP+FP.

## 3. Results

A total of 123320 PPIs, which are composed of 15214 cancer PPIs and 108106 noncancer PPIs, 2863 cancer proteins, and 3970 domains were used in our experiment. A 10-fold cross validation test was conducted to determine the optimal threshold settings for each classifier. We assumed the “*C*-*C*” type PPI as a positive set and the rest as a negative set. According to our previous work [30], the balanced trained dataset usually has better performance than the unbalanced one; hence, the algorithms are trained with an equal size ratio of 1 : 1 for the positive and negative dataset. Since the sizes of the original positive and negative sets differ by a factor of about 6 (unbalanced learning set), to generate a balanced learning set, the 15214 positive target interactions (cancer PPIs) were kept, and a total of 15214 noncancer PPIs were randomly selected from the negative set. Later on, the above-mentioned seven statistical measures are determined. For comparison, the corresponding results of unbalanced dataset are listed in Supplementary File 1, Appendix Tables S1 to S5, in Supplementary Material available online at http://dx.doi.org/10.1155/2015/312047, where the performance of MCC and PPV is much worse due to the very large TN and very small TP. Therefore, the use of balanced datasets is more preferable.

### 3.1. Performance Comparison by Individual Machine Learning Algorithm and Voting with the Majority

The performance comparison for the individual algorithm is listed in Table 1. The LMT algorithm of the “trees” type achieved the highest ACC (0.772), *F*1 (0.774), and MCC (0.548) among the 39 algorithms. Interestingly, according to either ACC, *F*1, or MCC, the top six algorithms (LMT, SimpleCart, J48, J48graft, REPTree, and FT) are all of the “trees” type. On the other hand, the LWL algorithm of the “lazy” type, the VFI algorithm of the “misc” type, the ConjunctiveRule algorithm of the “rules” type, and the DecisionStump algorithm of the “trees” type achieved the highest SPE and PPV (1.000), and the Nnge algorithm of the “rules” type achieved the highest SEN (0.858), while the Ridor algorithm of the “rules” type achieved the highest AUC (0.780). We also tried to combine the subsets of the four features for predicting the cancer proteins, but the predictive performance is not markedly improved.

The individual classifier has its own strengths and weaknesses; therefore, it is inspired to integrate multiple classifiers, that is, voting with the majority system, to improve the classification performance. Thirty-nine machine learning algorithms in Weka were selected and integrated using various types of voting with the majority system. In this study, the voting with the majority system involved an odd number of algorithms. For example, top 5 in Table 2 represents combining the top 5 algorithms extracted from Table 1, which are LMT, SimpleCart, J48, J48graft, and REPTree. Performance comparison by voting with the majority is listed in Table 2. The best performance in voting is attained when the top 23 algorithms are selected, which have the highest *F*1 (0.786) and PPV (0.890). When comparing Table 1 with Table 2, except SEN, the top voting with the majority system (top 23) is better than top individual algorithm (LMT) in the other six performance measures, and it also outperformed all thirty-nine individual algorithm in ACC, *F*1, MCC, and AUC. We also noted that the best performance of voting with the majority system (top 23) delivered lower FP events, that is, 186, than those of the top three individual algorithms, which are 303, 297, and 307, respectively. In other words, the voting approach does not introduce spurious events.

### 3.2. Performance Comparison with Group Voting with the Majority

Performance comparison by voting with the majority for each group is listed in Table 3 (Misc type is omitted here, because it contains only two algorithms). For instance, under the “functions” type, the “Top: 3” classifier in Table 3 represents the combination of “functions” type algorithms, that is, MultilayerPerceptron, Logistic, and SimpleLogistic algorithms (see Table 1). The results indicated that “trees” type achieved the highest ACC (0.781), SEN (0.749), *F*1 (0.786), MCC (0.567), MCC (0.513), and AUC (0.788), while the “rules” type had the highest SPE (0.838), and “lazy” type achieved the highest PPV (0.883).

**Figure 4:**
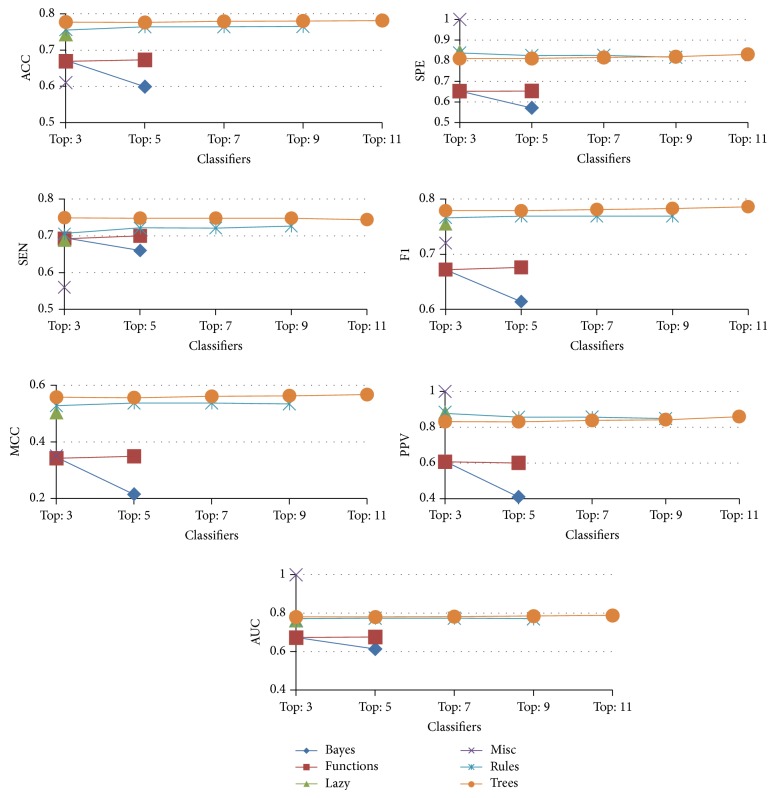
The seven performance measures for group voting with the majority.

For clarity, Figure 4 illustrates the majority voting results for various types of algorithms; the results suggest that “trees” type algorithms perform better in most of the performance measures.

### 3.3. Performance Comparison with the Competing Study

Since there are four features that are considered in this study, it is necessary to study their significance in classification performance. To study the prediction performance of the four features individually, we evaluated the feature importance by the area under the curve (AUC) value [36, 37]. Features with a higher AUC score are ranked as more important than features with a low score. The results of AUC values for the four features are given in Table 4. The DFS_C feature ranks at the top in AUC value, while the DFS_X feature has the lowest AUC value. These results suggest that DFS_C feature has the greatest discrimination information between positive datasets and negative datasets.

**Figure 5:**
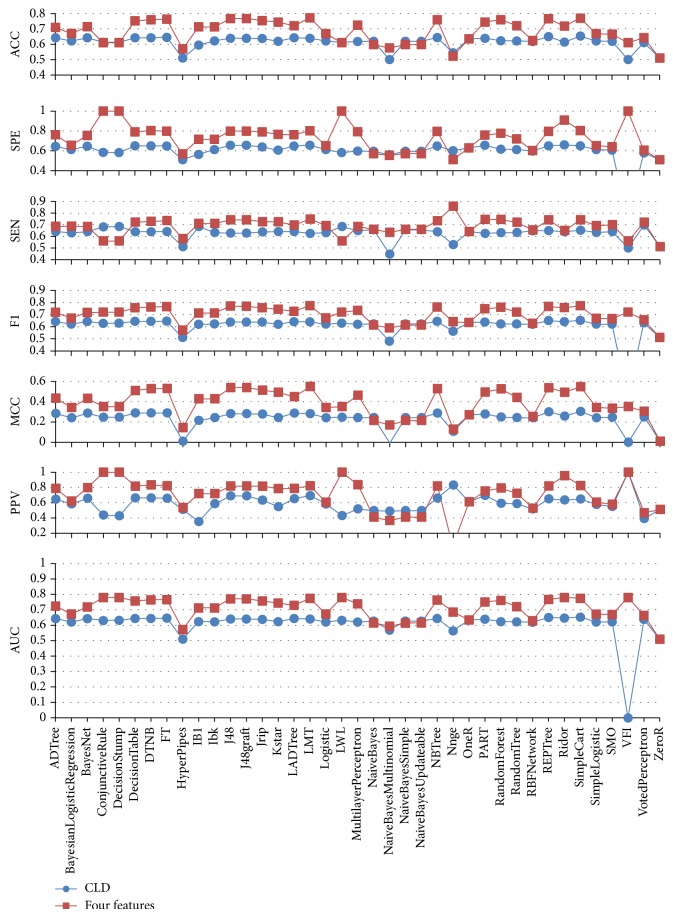
The performance comparison of the individual algorithm for Aragues (blue) and the proposed method (red).

To demonstrate the effectiveness of the present study, we compared our results with the work by Aragues et al. [4], which uses the CLD feature only. As shown in Table 5 and Figure 5, for a single classifier, our method achieved better performance than Aragues's in all seven performance measures. From Tables 1 and 5, we can see that the best single classifier of the current method (LMT) outperforms the best single classifier of Aragues's method (SimpleCart) in all seven performance measures; the difference value of ACC is 11.9%, SPE is 15.1%, SEN is 9.6%, *F*1 is 12.1%, MCC is 24.5%, PPV is 17.4%, and AUC is 12.1%.

**Figure 6:**
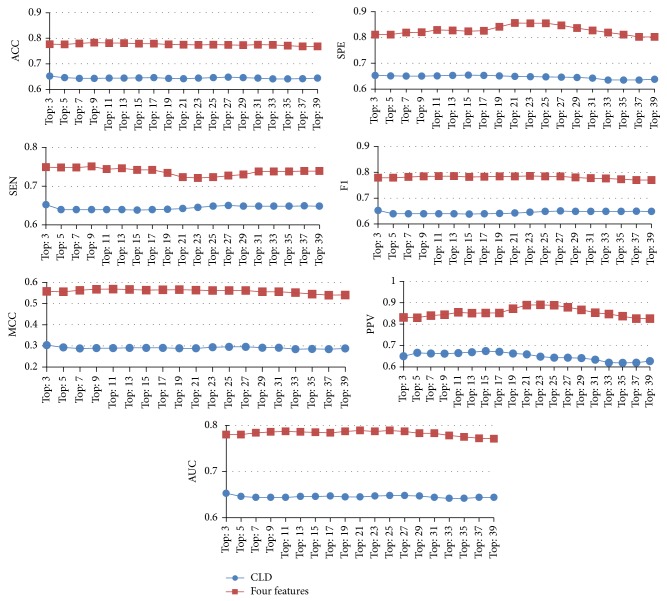
The performance comparison of the voting with the majority for Aragues (blue) and the proposed method (red).

From Tables 5 and 6, we can see that, using the CLD feature only, the best performance in voting is attained when the top 3 algorithms (SimpleCart, REPTree, and FT) are selected using the CLD feature only, but the performance of voting with the majority is approximately equal to that of the individual algorithm. As shown in Table 6 and Figure 6, the proposed method significantly outperformed Aragues's in all seven performance measures. From Tables 2 and 6, we can see that the best voting with the majority of the current method (top 23) outperforms the best voting with the majority of Aragues's method (top 3) in all seven performance measures; the difference value of ACC is 12.2%, SPE is 20.2%, SEN is 6.9%, *F*1 is 13.4%, MCC is 25.8%, PPV is 24.0%, and AUC is 13.4%. These results suggest that the proposed method is superior to the competing study.

## 4. Discussion

In this section, we designed two case studies to demonstrate the performance of the proposed method and showed how to discover potential cancer proteins, respectively.

### 4.1. Case Study 1

To investigate the performance of the proposed method, we retrieved lung cancer protein data from OMIM and HLungDB databases. Among the 2599 experimentally confirmed lung cancer proteins, there are a total of 1302 cancer proteins not appearing in our original training dataset, which could be used as the independent test dataset. List of the 1302 cancer proteins can be found in Supplementary File 2. The LWL, VFI, ConjunctiveRule, and DecisionStump were excluded from Case Study 1 because of their intrinsically high PPV which may bias the performance estimation. Consequently, as shown in Table 7, the hit number and hit ratio denote how many cancer proteins are true positive events and true positive ratios, respectively. Most of the algorithms had a hit ratio remarkably consistent over 75%, especially, the Ridor algorithm which achieved 89.4%. Compared with classifiers with higher ranks in *F*1 (Table 1), the results appear to suggest that classifiers with high PPV achieve better hit ratios.

### 4.2. Case Study 2

It is known that interacting proteins are often coexpressed; one can identify differentially expressed genes (DEGs) among a large number of gene expressions and understand the mechanism of lung cancer formation induced by these DEGs [38]. We further explored the potential cancer genes from DEGs in microarray data. Four sets of lung cancer microarray data were downloaded from the GEO database [39] and summarized in Table 8. Experiments GSE7670 [40] and GSE10072 [41] use the HG-U133A array, where GSE19804 [42] and GSE27262 [43] use HG-U133 plus 2.0 chip. The tested DEGs are collected from the intersection set of the above four microarray datasets. Among the 1345 common DEGs in the four microarray datasets, 360 DEGs were excluded because of their appearance in the original training set; another 209 DEGs are also removed due to their lacking of domain data or PPI data. The remaining 776 DEGs serve as input data.

Five classifiers, including the top three classifiers according to the *F*1 measure, LMT, SimpleCart, and J48 algorithms, as well as the top one classifier according to PPV measure, LWL algorithm, along with the top one classifier according to Case Study 1, Ridor algorithm, were selected for evaluating potential cancer genes under strictly uniformed voting; that is, only the one with five votes which all five classifiers predict as a cancer protein was considered. Among the 776 DEGs, a total of 565 DEGs (72.8%, a higher value can be obtained if we relax the strictly uniformed requirement) were evaluated as potential cancer genes. The complete 565 potential cancer genes derived from the second case study are listed in Supplementary File 3.

To validate our findings, we conducted a study of the literature by randomly selecting five genes (DBF4, MCM2, ID3, EXOSC4, and CDKN3) from the 565 potential cancer genes. The results indicated that while EXOSC4 remains unclear, the others are in agreement with our predictions. Barkley proposed that miR-29a targets the 3-UTR of DBF4 mRNA in lung cancer cells [44] and Bonte stated that most cell lines with increased Cdc7 protein levels also had increased DBF4 abundance, and some tumor cell lines had extra copies of the DBF4 gene [45]. Alexandrow noted that Stat3-P and the proliferative markers MCM2 were expressed in mice lung tissues* in vivo* [46]. Yang et al. observed that patients with higher levels of MCM2 and gelsolin experienced shorter survival time than patients with low levels of MCM2 and gelsolin [47]. Langenfeld et al. [48] indicated that Oct4 cells give rise to lung cancer cells expressing nestin and/or NeuN, and BMP signaling is an important regulator of ID1 and ID3 in both Oct4 and nestin cell populations. Tang claimed that CDKN3 has significant biological implications in tumor pathogenesis [49]. In [50], a metasignature was identified in eight separate microarray analyses spanning seven types of cancer including lung adenocarcinoma, and these included many genes associated with cell proliferation, and CDKN3 is among them.

Given a single protein as testing data, we can first treat this protein as *X*. If both PPIs and domains information are available, one can then apply the present method to classify the interaction type “*C*-*X*”.

Any “*C*-*X*” type is classified as “*C*-*C*”, and then there are two possible explanations for this: (i) the classifier is not completely specific; therefore, one has FP prediction, and (ii) prediction is a TP event. If one can exclude the first explanation option, then the present calculation provides a potential way to assign *X* as *C*; in other words, it provides a feasible solution for predicting cancer proteins.

If the PPI information is missing, given the FASTA sequence information, one can make use of the STRING database [51]. STRING is a database that provides known and predicted PPI derived from four sources: genomic context, high-throughput experiments, conserved coexpression, and published literature. On domain prediction, one can carry out the analysis by using the online tool “SEQUENCE SEARCH” under PFam [34] to find matching domains. Then, given the PPIs and domains information, one can conduct the same analysis as described in the last paragraph; otherwise, one is facing a difficult task, which requires further discussion or work.

## 5. Conclusion

Identifying cancer protein is a critical issue in treating cancer; however, identifying cancer protein experimentally is extremely time consuming and labor-intensive. Alternative methods must be developed to discover cancer proteins. We have integrated several proteomic data sources to develop a model for predicting cancer protein-cancer protein interactions on a global scale based on domain-domain interactions, weighted domain frequency score, and cancer linker degree. A one-to-one interaction model was introduced to quantify the likelihood of cancer-specific DDI. The weighted DFS is adopted to measure the propensity of domain occurrence in cancer and noncancer proteins. Finally, the CLD is defined to gauge cancer and noncancer proteins' interaction partners. As a result, voting with a majority system achieved ACC (0.774), SPE (0.855), SEN (0.721), *F*1 (0.786), MCC (0.562), PPV (0.890), and AUC (0.787) when the top 23 algorithms were selected, which is better than the best single classifier (LMT) in six performance measures except SEN.

We compared our performance with the previous work [4]. It was shown that the present approach outperformed Aragues's in all seven performance measures in both individual algorithm and combining algorithms. Effectiveness of the current research is further evaluated by two independent datasets; experimental results demonstrated that the proposed method can identify cancer proteins with high hit ratios. The current research not only significantly improves the prediction performance of cancer proteins, but also discovered some potential cancer proteins for future experimental investigation. It is anticipated that the current research could provide some insight into disease mechanisms and diagnosis.

## Supplementary Material

Supplementary File 1: Experimental results with unbalanced data. Here, the corresponding experimental results of the unbalanced dataset are listed in Appendix Tables S1 to S5, where the performance of MCC and PPV is much worse due to the very large TN and very small TP. Therefore, the use of balanced datasets is more preferable.
Supplementary File 2: List of the 1302 cancer proteins for Case Study 1. List of the 1302 cancer proteins extracted from the OMIM and HLungDB data-bases, that are not appear in our original training dataset. The 1302 cancer pro-teins are used as an independent test dataset for Case Study 1.
Supplementary File 3: List of the 565 potential cancer genes derived from Case Study 2. List of the 565 potential cancer genes derived from four sets of lung cancer mi-croarray data by our method. Five classifiers, including LMT, SimpleCart, J48, LWL and Ridor algorithms were selected for evaluating potential cancer genes under strictly uniformed voting; that is, only the one with five votes which all five classifiers predict as a cancer protein was considered. The 565 potential cancer genes are good targets for future experimental investigation.

## Figures and Tables

**Table 1:** Performance comparison for the individual algorithm sorted by *F*1 value.

Type	Algorithm	ACC	SPE	SEN	*F*1	MCC	PPV	AUC
Trees	LMT	0.772	0.802	0.748	0.774	0.548	0.821	0.774
Trees	SimpleCart	0.770	0.804	0.742	0.773	0.546	0.825	0.775
Trees	J48	0.767	0.799	0.741	0.770	0.538	0.818	0.771
Trees	J48graft	0.767	0.799	0.742	0.769	0.538	0.818	0.771
Trees	REPTree	0.766	0.796	0.741	0.767	0.536	0.818	0.767
Trees	FT	0.763	0.798	0.735	0.766	0.528	0.821	0.766
Rules	DTNB	0.760	0.804	0.728	0.763	0.527	0.833	0.765
Trees	NBTree	0.760	0.796	0.733	0.763	0.527	0.819	0.764
Trees	RandomForest	0.760	0.777	0.744	0.761	0.524	0.791	0.761
Rules	Ridor	0.718	0.910	0.650	0.758	0.492	0.954	0.780
Rules	Jrip	0.754	0.790	0.726	0.757	0.512	0.817	0.757
Rules	DecisionTable	0.752	0.790	0.722	0.756	0.509	0.817	0.757
Rules	PART	0.744	0.758	0.745	0.748	0.494	0.754	0.751
Lazy	Kstar	0.744	0.766	0.725	0.744	0.491	0.784	0.744
Functions	MultilayerPerceptron	0.724	0.792	0.683	0.734	0.462	0.835	0.739
Trees	LADTree	0.720	0.762	0.696	0.727	0.447	0.788	0.729
Trees	RandomTree	0.721	0.721	0.720	0.721	0.440	0.723	0.721
Lazy	LWL	0.610	1.000	0.560	0.720	0.350	1.000	0.780
Misc	VFI	0.610	1.000	0.560	0.720	0.350	1.000	0.780
Rules	ConjunctiveRule	0.610	1.000	0.560	0.720	0.350	1.000	0.780
Trees	DecisionStump	0.610	1.000	0.560	0.720	0.350	1.000	0.780
Trees	ADTree	0.709	0.762	0.685	0.719	0.433	0.787	0.724
Bayes	BayesNet	0.714	0.755	0.683	0.717	0.431	0.796	0.719
Lazy	IB1	0.713	0.716	0.711	0.713	0.427	0.718	0.713
Lazy	Ibk	0.713	0.716	0.711	0.713	0.427	0.718	0.713
Functions	Logistic	0.669	0.652	0.692	0.672	0.342	0.606	0.673
Bayes	BayesianLogisticRegression	0.670	0.655	0.687	0.671	0.342	0.622	0.673
Functions	SimpleLogistic	0.669	0.652	0.691	0.670	0.342	0.605	0.672
Functions	SMO	0.665	0.641	0.699	0.667	0.334	0.580	0.670
Functions	VotedPerceptron	0.642	0.606	0.721	0.657	0.305	0.467	0.664
Rules	Nnge	0.522	0.511	0.858	0.641	0.128	0.052	0.686
Rules	OneR	0.635	0.629	0.641	0.635	0.269	0.610	0.634
Functions	RBFNetwork	0.624	0.603	0.655	0.627	0.254	0.529	0.629
Bayes	NaiveBayes	0.598	0.571	0.660	0.614	0.214	0.410	0.615
Bayes	NaiveBayesUpdateable	0.598	0.571	0.660	0.614	0.214	0.410	0.615
Bayes	NaiveBayesSimple	0.598	0.571	0.660	0.613	0.214	0.411	0.614
Bayes	NaiveBayesMultinomial	0.576	0.554	0.634	0.590	0.168	0.366	0.594
Misc	HyperPipes	0.570	0.568	0.579	0.571	0.143	0.534	0.572
Rules	ZeroR	0.510	0.510	0.510	0.510	0.010	0.510	0.510

**Table 2:** Performance comparison by voting with the majority sorted by *F*1 value.

Classifiers	ACC	SPE	SEN	*F*1	MCC	PPV	AUC
TOP: 23	0.774	0.855	0.721	0.786	0.562	0.890	0.787
TOP: 11	0.781	0.829	0.744	0.785	0.569	0.855	0.787
TOP: 13	0.781	0.827	0.746	0.785	0.567	0.851	0.786
TOP: 9	0.783	0.820	0.751	0.784	0.568	0.844	0.786
TOP: 19	0.776	0.841	0.734	0.784	0.566	0.872	0.787
TOP: 21	0.775	0.856	0.723	0.784	0.564	0.889	0.789
TOP: 25	0.775	0.855	0.723	0.784	0.562	0.888	0.789
TOP: 27	0.774	0.847	0.727	0.784	0.562	0.879	0.787
TOP: 17	0.779	0.826	0.742	0.783	0.565	0.852	0.784
TOP: 7	0.780	0.819	0.748	0.782	0.563	0.840	0.784
TOP: 15	0.779	0.824	0.742	0.782	0.564	0.852	0.785
TOP: 29	0.773	0.836	0.730	0.780	0.556	0.867	0.783
TOP: 3	0.777	0.811	0.749	0.779	0.558	0.831	0.780
TOP: 5	0.776	0.811	0.748	0.779	0.556	0.830	0.780
TOP: 31	0.775	0.827	0.738	0.777	0.556	0.853	0.783
TOP: 33	0.774	0.819	0.738	0.776	0.552	0.847	0.778
TOP: 35	0.771	0.811	0.738	0.773	0.545	0.837	0.775
TOP: 37	0.768	0.802	0.739	0.770	0.540	0.826	0.772
TOP: 39	0.768	0.802	0.739	0.770	0.541	0.826	0.771

**Table 3:** Performance comparison by voting with the majority for five classification types.

Type	Classifiers	ACC	SPE	SEN	*F*1	MCC	PPV	AUC
Bayes	TOP: 3	0.672	0.653	0.695	0.672	0.346	0.609	0.673
	TOP: 5	0.599	0.571	0.660	0.614	0.215	0.410	0.614

Functions	TOP: 5	0.673	0.653	0.700	0.676	0.349	0.600	0.676
	TOP: 3	0.669	0.652	0.692	0.672	0.342	0.606	0.673

Lazy	TOP: 3	0.743	0.837	0.688	0.755	0.504	0.883	0.763

Rules	TOP: 5	0.764	0.825	0.721	0.769	0.537	0.856	0.774
	TOP: 7	0.764	0.826	0.721	0.769	0.537	0.857	0.774
	TOP: 9	0.765	0.817	0.726	0.769	0.534	0.848	0.771
	TOP: 3	0.755	0.838	0.705	0.766	0.528	0.877	0.771

Trees	TOP: 11	0.781	0.831	0.744	0.786	0.567	0.859	0.788
	TOP: 9	0.780	0.820	0.748	0.783	0.563	0.842	0.785
	TOP: 7	0.779	0.816	0.748	0.781	0.561	0.838	0.782
	TOP: 3	0.777	0.811	0.749	0.779	0.558	0.831	0.780
	TOP: 5	0.776	0.811	0.748	0.779	0.556	0.830	0.780

**Table 4:** The AUC value of the four features.

Feature	AUC	Rank
DFS_C	0.677	1
CLD	0.651	2
DDI	0.546	3
DFS_X	0.526	4

**Table 5:** Performance comparison for the individual algorithm using the CLD feature sorted by *F*1.

Type	Algorithm	ACC	SPE	SEN	*F*1	MCC	PPV	AUC
Trees	SimpleCart	0.653	0.651	0.652	0.653	0.303	0.647	0.653
Trees	REPTree	0.650	0.650	0.649	0.650	0.300	0.649	0.650
Trees	FT	0.645	0.649	0.641	0.646	0.288	0.658	0.646
Rules	DecisionTable	0.642	0.650	0.639	0.644	0.288	0.663	0.644
Rules	DTNB	0.642	0.650	0.639	0.644	0.288	0.663	0.644
Trees	NBTree	0.643	0.648	0.639	0.644	0.287	0.661	0.644
Bayes	BayesNet	0.642	0.647	0.640	0.643	0.286	0.657	0.643
Trees	ADTree	0.642	0.644	0.641	0.643	0.283	0.646	0.643
Rules	Ridor	0.614	0.659	0.639	0.642	0.257	0.635	0.647
Trees	LADTree	0.642	0.648	0.641	0.642	0.287	0.653	0.644
Trees	LMT	0.639	0.656	0.625	0.640	0.280	0.693	0.640
Rules	PART	0.639	0.655	0.625	0.639	0.278	0.695	0.639
Trees	J48	0.639	0.655	0.627	0.639	0.280	0.689	0.641
Trees	J48graft	0.639	0.655	0.627	0.639	0.280	0.689	0.641
Rules	Jrip	0.637	0.639	0.638	0.638	0.277	0.634	0.638
Rules	OneR	0.635	0.629	0.641	0.635	0.269	0.610	0.634
Functions	VotedPerceptron	0.611	0.579	0.697	0.632	0.248	0.392	0.638
Lazy	LWL	0.612	0.582	0.683	0.629	0.246	0.431	0.632
Trees	DecisionStump	0.612	0.582	0.684	0.629	0.246	0.428	0.632
Rules	ConjunctiveRule	0.613	0.583	0.681	0.628	0.245	0.437	0.631
Bayes	NaiveBayes	0.619	0.595	0.656	0.623	0.242	0.496	0.627
Bayes	NaiveBayesSimple	0.619	0.595	0.656	0.623	0.242	0.496	0.627
Bayes	NaiveBayesUpdateable	0.619	0.595	0.656	0.623	0.242	0.496	0.627
Trees	RandomForest	0.623	0.616	0.631	0.623	0.247	0.591	0.624
Lazy	Ibk	0.622	0.613	0.632	0.622	0.242	0.586	0.622
Trees	RandomTree	0.622	0.613	0.632	0.622	0.242	0.586	0.622
Bayes	BayesianLogisticRegression	0.621	0.612	0.630	0.621	0.240	0.583	0.621
Functions	Logistic	0.621	0.612	0.631	0.621	0.241	0.582	0.621
Functions	SimpleLogistic	0.621	0.612	0.633	0.621	0.241	0.578	0.621
Functions	SMO	0.619	0.607	0.640	0.621	0.245	0.549	0.622
Lazy	Kstar	0.619	0.606	0.640	0.620	0.242	0.547	0.623
Functions	MultilayerPerceptron	0.618	0.598	0.649	0.620	0.242	0.518	0.621
Functions	RBFNetwork	0.618	0.598	0.647	0.620	0.240	0.517	0.620
Lazy	IB1	0.594	0.564	0.683	0.619	0.214	0.351	0.624
Rules	Nnge	0.544	0.601	0.529	0.562	0.106	0.832	0.564
Misc	HyperPipes	0.510	0.510	0.510	0.510	0.010	0.510	0.510
Rules	ZeroR	0.510	0.510	0.510	0.510	0.010	0.510	0.510
Bayes	NaiveBayesMultinomial	0.500	0.561	0.447	0.480	−0.008	0.489	0.569
Misc	VFI	0.500	NA	0.500	NA	NA	1.000	NA

**Table 6:** Performance comparison by voting with the majority using the CLD feature sorted by *F*1.

Classifiers	ACC	SPE	SEN	*F*1	MCC	PPV	AUC
TOP: 3	0.652	0.653	0.652	0.652	0.304	0.650	0.653
TOP: 27	0.648	0.646	0.650	0.648	0.296	0.643	0.648
TOP: 17	0.646	0.653	0.639	0.647	0.291	0.671	0.647
TOP: 25	0.646	0.647	0.648	0.647	0.296	0.643	0.648
TOP: 5	0.646	0.651	0.639	0.646	0.293	0.666	0.646
TOP: 15	0.645	0.654	0.638	0.646	0.291	0.674	0.646
TOP: 19	0.643	0.651	0.640	0.646	0.289	0.663	0.645
TOP: 23	0.644	0.648	0.645	0.646	0.294	0.648	0.647
TOP: 29	0.646	0.645	0.648	0.646	0.292	0.641	0.647
TOP: 13	0.644	0.653	0.639	0.645	0.291	0.669	0.646
TOP: 31	0.644	0.642	0.648	0.645	0.292	0.634	0.644
TOP: 7	0.643	0.650	0.639	0.644	0.288	0.663	0.644
TOP: 9	0.643	0.650	0.639	0.644	0.290	0.662	0.644
TOP: 11	0.644	0.651	0.639	0.644	0.290	0.665	0.644
TOP: 21	0.642	0.649	0.642	0.644	0.289	0.659	0.645
TOP: 39	0.644	0.638	0.648	0.644	0.288	0.627	0.644
TOP: 37	0.642	0.635	0.649	0.643	0.285	0.620	0.644
TOP: 35	0.641	0.635	0.648	0.642	0.286	0.619	0.642
TOP: 33	0.641	0.635	0.648	0.641	0.285	0.620	0.642

**Table 7:** Performance evaluation for OMIM and HLungDB datasets.

Type	Algorithm	Hit number	Hit ratio
Rules	Ridor	1164	0.894
Rules	ZeroR	1159	0.890
Trees	ADTree	1119	0.859
Misc	HyperPipes	1115	0.856
Functions	MultilayerPerceptron	1076	0.826
Trees	LADTree	1061	0.815
Rules	OneR	1047	0.804
Lazy	IB1	1023	0.786
Lazy	IBk	1023	0.786
Bayes	BayesNet	1020	0.783
Rules	PART	1019	0.783
Trees	J48graft	1018	0.782
Trees	J48	1017	0.781
Rules	DecisionTable	1011	0.776
Trees	FT	1004	0.771
Lazy	KStar	999	0.767
Bayes	BayesianLogisticRegression	998	0.767
Trees	NBTree	995	0.764
Rules	DTNB	991	0.761
Trees	RandomTree	988	0.759
Trees	LMT	983	0.755
Trees	REPTree	975	0.749
Trees	SimpleCart	973	0.747
Trees	RandomForest	973	0.747
Rules	JRip	966	0.742
Functions	Logistic	964	0.740
Functions	SimpleLogistic	964	0.740
Functions	SMO	944	0.725
Functions	RBFNetwork	925	0.710
Functions	VotedPerceptron	844	0.648
Bayes	NaiveBayesSimple	827	0.635
Bayes	NaiveBayes	826	0.634
Bayes	NaiveBayesUpdateable	826	0.634
Bayes	NaiveBayesMultinomial	796	0.611
Rules	NNge	94	0.072

**Table 8:** Summary of microarray datasets.

GEO ID	Organization name	Number of DEGs
GSE7670	Taipei Veterans General Hospital	1874
GSE10072	National Cancer Institute, NIH	3138
GSE19804	National Taiwan University	5398
GSE27262	National Taiwan Yang Ming University	8476
		1345 (intersection)
