# Supplementary material for: Prediction of Cancer Proteins by Integrating Protein Interaction, Domain Frequency, and Domain Interaction Data Using Machine Learning Algorithms
Source: Biomed Res Int. 2015 Mar 17;2015:312047. doi: 10.1155/2015/312047 (PMC4381656; doi:10.1155/2015/312047)
Supplement: Supplementary file 1 — Experimental results with unbalanced data. Here, the corresponding experimental results of the unbalanced dataset are listed in Appendix Tables S1 to S5, where the performance of MCC and PPV is much worse due to the very large TN and very small TP. Therefore, the use of balanced datasets is more preferable. Supplementary File 2: List of the 1302 cancer proteins for Case Study 1. List of the 1302 cancer proteins extracted from the OMIM and HLungDB data-bases, that are not appear in our original training dataset. The 1302 cancer pro-teins are used as an independent test dataset for Case Study 1. Supplementary File 3: List of the 565 potential cancer genes derived from Case Study 2. List of the 565 potential cancer genes derived from four sets of lung cancer mi-croarray data by our method. Five classifiers, including LMT, SimpleCart, J48, LWL and Ridor algorithms were selected for evaluating potential cancer genes under strictly uniformed voting; that is, only the one with five votes which all five classifiers predict as a cancer protein was considered. The 565 potential cancer genes are good targets for future experimental investigation. [file 312047.f1.zip › 312047.f1/paper-cancer-PPI-suppl-files/Supplementary File 1_Appendix_20150225.pdf]

Appendix – Experimental results with unbalanced data

TABLE S1. Performance comparison for the individual algorithm sorted by F1 value (Unbalanced data).

Type	Algorithm	ACC	SPE	SEN	F1	MCC	PPV	AUC
lazy	Kstar	0.865	0.865	0.944	0.903	0.223	0.062	0.904
rules	Ridor	0.862	0.862	0.847	0.854	0.172	0.043	0.855
trees	REPTree	0.894	0.903	0.765	0.828	0.483	0.368	0.834
trees	LADTree	0.866	0.867	0.785	0.824	0.225	0.082	0.826
trees	J48	0.886	0.893	0.763	0.823	0.426	0.291	0.828
trees	J48graft	0.886	0.893	0.764	0.823	0.424	0.289	0.828
trees	FT	0.882	0.888	0.761	0.820	0.394	0.252	0.824
trees	SimpleCart	0.883	0.891	0.741	0.809	0.408	0.280	0.816
rules	PART	0.877	0.882	0.746	0.808	0.353	0.210	0.814
trees	ADTree	0.865	0.867	0.729	0.792	0.222	0.088	0.798
trees	NBTree	0.875	0.883	0.716	0.791	0.345	0.213	0.799
rules	OneR	0.866	0.871	0.679	0.763	0.250	0.123	0.775
rules	DecisionTable	0.879	0.896	0.655	0.757	0.404	0.325	0.775
rules	Jrip	0.865	0.869	0.664	0.753	0.229	0.107	0.767
rules	DTNB	0.870	0.880	0.641	0.742	0.308	0.200	0.761
functions	MultilayerPerceptron	0.859	0.862	0.607	0.712	0.136	0.044	0.735
bayes	BayesNet	0.850	0.885	0.459	0.604	0.268	0.260	0.672
functions	Logistic	0.853	0.860	0.357	0.505	0.069	0.031	0.608
functions	VotedPerceptron	0.849	0.863	0.354	0.502	0.099	0.066	0.608
bayes	BayesianLogisticRegression	0.853	0.860	0.344	0.491	0.068	0.033	0.602
functions	RBFNetwork	0.856	0.858	0.324	0.470	0.027	0.006	0.591
trees	LMT	0.794	0.883	0.290	0.437	0.177	0.306	0.586
bayes	NaiveBayes	0.816	0.867	0.254	0.393	0.095	0.147	0.561
bayes	NaiveBayesUpdateable	0.816	0.867	0.254	0.393	0.095	0.147	0.561
bayes	NaiveBayesSimple	0.815	0.867	0.251	0.389	0.094	0.148	0.559
misc	HyperPipes	0.597	0.885	0.183	0.303	0.096	0.526	0.534
misc	VFI	0.329	1.000	0.176	0.299	0.195	1.000	0.588
functions	SimpleLogistic	0.501	0.858	0.144	0.247	0.003	0.504	0.501
trees	RandomTree	0.752	0.857	0.143	0.245	0.000	0.146	0.500
lazy	IB1	0.755	0.857	0.140	0.241	-0.003	0.139	0.501

lazy	Ibk	0.755	0.857	0.140	0.241	-0.003	0.139	0.501
trees	RandomForest	0.800	0.856	0.135	0.233	-0.007	0.073	0.505
bayes	NaiveBayesMultinomial	0.857	0.857	NA	NA	NA	0.000	NA
functions	SMO	0.857	0.857	NA	NA	NA	0.000	NA
lazy	LWL	0.857	0.857	NA	NA	NA	0.000	NA
rules	ConjunctiveRule	0.857	0.857	NA	NA	NA	0.000	NA
rules	ZeroR	0.857	0.857	NA	NA	NA	0.000	NA
trees	DecisionStump	0.857	0.857	NA	NA	NA	0.000	NA
rules	Nnge	NA	NA	NA	NA	NA	NA	NA

TABLE S2. Performance comparison by voting with the majority sorted by F1 value (Unbalanced data).

Classifiers	ACC	SPE	SEN	F1	MCC	PPV	AUC
TOP: 37	0.862	0.861	0.967	0.911	0.165	0.033	0.914
TOP: 35	0.863	0.863	0.951	0.905	0.194	0.047	0.907
TOP: 33	0.865	0.865	0.930	0.896	0.221	0.062	0.898
TOP: 25	0.869	0.869	0.922	0.895	0.271	0.095	0.895
TOP: 31	0.868	0.867	0.919	0.892	0.255	0.084	0.893
TOP: 5	0.871	0.870	0.908	0.889	0.289	0.109	0.889
TOP: 3	0.866	0.866	0.911	0.888	0.233	0.071	0.888
TOP: 21	0.870	0.870	0.907	0.888	0.281	0.104	0.888
TOP: 23	0.871	0.87	0.906	0.888	0.283	0.105	0.888
TOP: 29	0.870	0.869	0.903	0.886	0.276	0.101	0.886
TOP: 27	0.870	0.870	0.900	0.885	0.280	0.104	0.885
TOP: 19	0.873	0.872	0.891	0.881	0.306	0.125	0.882
TOP: 17	0.876	0.875	0.881	0.878	0.333	0.150	0.878
TOP: 13	0.879	0.880	0.873	0.876	0.366	0.183	0.876
TOP: 15	0.878	0.878	0.870	0.874	0.353	0.171	0.874
TOP: 7	0.881	0.882	0.861	0.871	0.379	0.199	0.871
TOP: 9	0.881	0.883	0.846	0.864	0.383	0.207	0.864
TOP: 11	0.879	0.880	0.847	0.863	0.365	0.190	0.863

TABLE S3. Performance comparison by voting with the majority for five classification types (Unbalanced data).

Type	Classifiers	ACC	SPE	SEN	F1	MCC	PPV	AUC
Bayes	TOP: 3	0.845	0.866	0.351	0.500	0.123	0.101	0.608
	TOP: 5	0.816	0.867	0.254	0.393	0.095	0.147	0.561
functions	TOP: 5	0.856	0.859	0.395	0.541	0.059	0.018	0.627
	TOP: 3	0.854	0.860	0.362	0.510	0.069	0.030	0.611
lazy	TOP: 3	0.755	0.857	0.14	0.241	-0.003	0.139	0.501
rules	TOP: 3	0.866	0.866	0.905	0.885	0.230	0.070	0.885
	TOP: 7	0.866	0.866	0.861	0.863	0.227	0.073	0.863
	TOP: 5	0.869	0.869	0.850	0.859	0.265	0.101	0.860
trees	TOP: 11	0.881	0.882	0.843	0.862	0.380	0.206	0.863
	TOP: 3	0.885	0.887	0.820	0.852	0.410	0.246	0.854
	TOP: 7	0.883	0.885	0.821	0.852	0.394	0.228	0.853
	TOP: 9	0.883	0.886	0.817	0.850	0.398	0.234	0.851
	TOP: 5	0.885	0.889	0.802	0.843	0.412	0.256	0.845

TABLE S4. Performance comparison for the individual algorithm using the *CLD* feature sorted by F1 (Unbalanced data).

Type	Algorithm	ACC	SPE	SEN	F1	MCC	PPV	AUC
bayes	BayesNet	0.858	0.858	1.000	0.924	0.076	0.007	0.929
trees	LADTree	0.858	0.858	1.000	0.924	0.076	0.007	0.929
trees	NBTree	0.858	0.858	1.000	0.924	0.076	0.007	0.929
trees	FT	0.859	0.859	0.996	0.922	0.110	0.014	0.927
rules	DecisionTable	0.859	0.858	0.954	0.903	0.097	0.012	0.906
rules	DTNB	0.859	0.858	0.954	0.903	0.097	0.012	0.906
rules	Ridor	0.857	0.857	0.917	0.886	0.022	0.001	0.887
trees	SimpleCart	0.859	0.859	0.892	0.875	0.117	0.019	0.875
lazy	Ibk	0.897	0.903	0.816	0.857	0.500	0.362	0.859
trees	RandomTree	0.897	0.903	0.816	0.857	0.500	0.362	0.859
trees	RandomForest	0.893	0.899	0.792	0.842	0.474	0.340	0.846
trees	REPTree	0.863	0.864	0.761	0.809	0.188	0.060	0.812
rules	OneR	0.866	0.871	0.679	0.763	0.250	0.123	0.775
rules	Jrip	0.857	0.857	0.610	0.713	0.029	0.002	0.734
rules	Nnge	0.855	0.907	0.491	0.637	0.377	0.431	0.699
lazy	IB1	0.841	0.911	0.448	0.601	0.367	0.472	0.680

functions	VotedPerceptron	0.849	0.860	0.296	0.440	0.064	0.043	0.578
bayes	NaiveBayes	0.851	0.859	0.290	0.434	0.051	0.029	0.575
bayes	NaiveBayesSimple	0.851	0.859	0.290	0.434	0.051	0.029	0.575
bayes	NaiveBayesUpdateable	0.851	0.859	0.290	0.434	0.051	0.029	0.575
functions	Logistic	0.856	0.857	0.274	0.415	0.017	0.004	0.566
bayes	BayesianLogisticRegression	0.856	0.857	0.268	0.408	0.012	0.002	0.563
functions	SimpleLogistic	0.501	0.858	0.144	0.247	0.003	0.504	0.501
misc	HyperPipes	0.501	0.858	0.144	0.247	0.003	0.504	0.501
trees	LMT	0.502	0.859	0.144	0.247	0.004	0.505	0.502
bayes	NaiveBayesMultinomial	0.857	0.857	NA	NA	NA	0.000	NA
functions	MultilayerPerceptron	0.857	0.857	NA	NA	NA	0.000.	NA
functions	RBFNetwork	0.857	0.857	NA	NA	NA	0.000	NA
functions	SMO	0.857	0.857	NA	NA	NA	0.000	NA
lazy	Kstar	0.857	0.857	NA	NA	NA	0.000	NA
lazy	LWL	0.857	0.857	NA	NA	NA	0.000	NA
misc	VFI	0.143	NA	0.143	NA	NA	1.000	NA
rules	ConjunctiveRule	0.857	0.857	NA	NA	NA	0.000	NA
rules	PART	0.857	0.857	NA	NA	NA	0.000	NA
rules	ZeroR	0.857	0.857	NA	NA	NA	0.000	NA
trees	ADTree	0.857	0.857	NA	NA	NA	0.000	NA
trees	DecisionStump	0.857	0.857	NA	NA	NA	0.000	NA
trees	J48	0.857	0.857	NA	NA	NA	0.000	NA
trees	J48graft	0.857	0.857	NA	NA	NA	0.000	NA

TABLE S5. Performance comparison by voting with the majority using the *CLD* feature sorted by F1 (Unbalanced data) .

Classifiers	ACC	SPE	SEN	F1	MCC	PPV	AUC
TOP: 3	0.858	0.858	1.000	0.924	0.076	0.007	0.929
TOP: 5	0.858	0.858	1.000	0.924	0.076	0.007	0.929
TOP: 7	0.858	0.858	1.000	0.924	0.076	0.007	0.929
TOP: 29	0.858	0.857	1.000	0.923	0.055	0.004	0.929
TOP: 31	0.858	0.857	1.000	0.923	0.054	0.003	0.929
TOP: 33	0.858	0.857	1.000	0.923	0.054	0.003	0.929
TOP: 35	0.858	0.857	1.000	0.923	0.054	0.003	0.929
TOP: 13	0.859	0.859	0.996	0.922	0.115	0.016	0.928

TOP: 15	0.859	0.859	0.996	0.922	0.115	0.016	0.928
TOP: 17	0.859	0.859	0.996	0.922	0.115	0.016	0.928
TOP: 9	0.859	0.858	0.995	0.921	0.095	0.011	0.927
TOP: 11	0.859	0.858	0.995	0.921	0.099	0.012	0.927
TOP: 19	0.859	0.859	0.982	0.916	0.102	0.012	0.920
TOP: 27	0.858	0.858	0.983	0.916	0.091	0.010	0.921
TOP: 21	0.859	0.858	0.977	0.914	0.099	0.012	0.918
TOP: 23	0.858	0.858	0.978	0.914	0.091	0.010	0.918
TOP: 25	0.859	0.858	0.950	0.902	0.097	0.012	0.904
TOP: 37	0.857	0.857	NA	NA	NA	0.000	NA
TOP: 39	0.857	0.857	NA	NA	NA	0.000	NA
