# Supplementary figures and images for: Prediction of Cancer Proteins by Integrating Protein Interaction, Domain Frequency, and Domain Interaction Data Using Machine Learning Algorithms
Source: Biomed Res Int. 2015 Mar 17;2015:312047. doi: 10.1155/2015/312047 (PMC4381656; doi:10.1155/2015/312047)

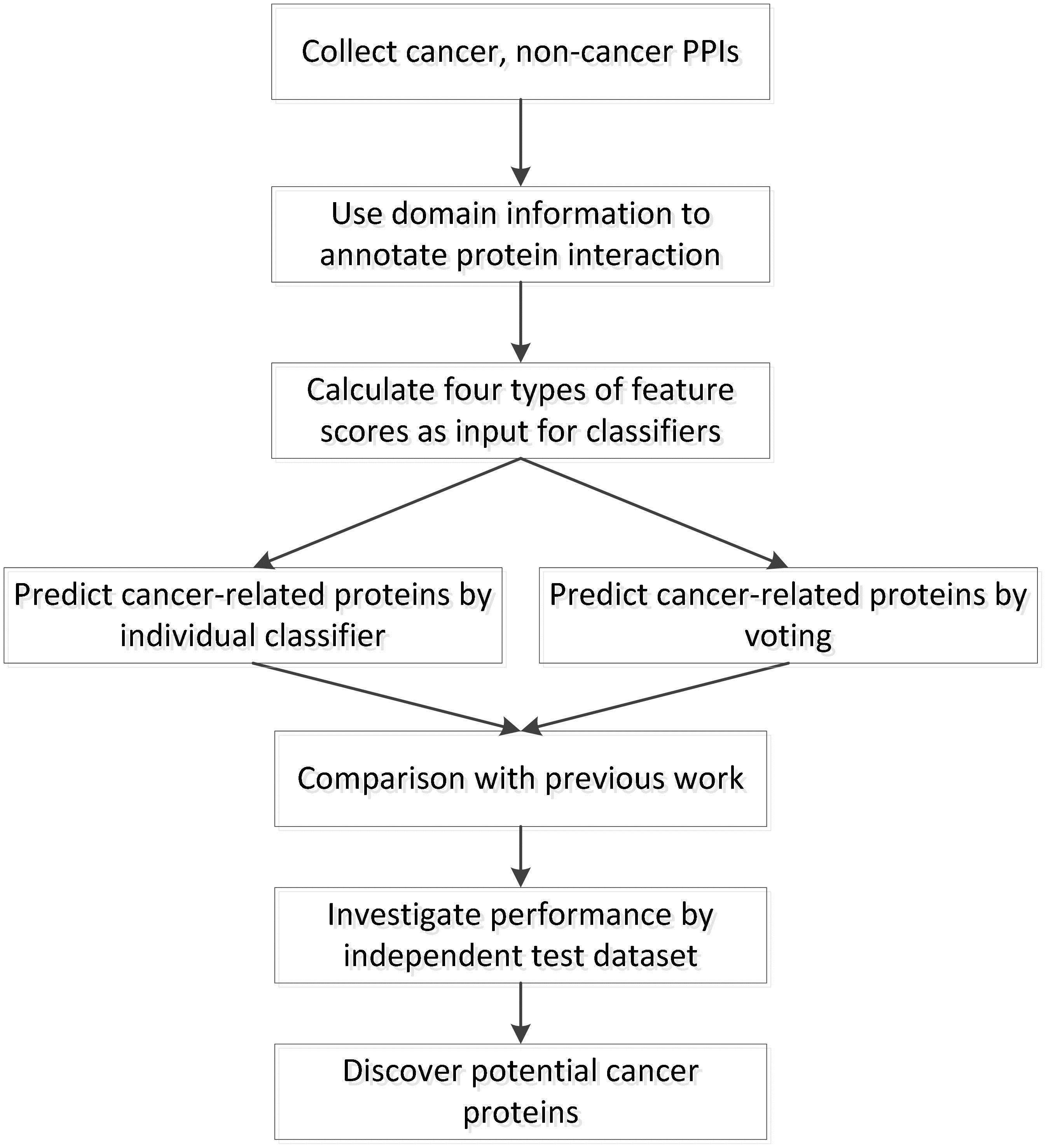

Supplement: Supplementary file 1 — Experimental results with unbalanced data. Here, the corresponding experimental results of the unbalanced dataset are listed in Appendix Tables S1 to S5, where the performance of MCC and PPV is much worse due to the very large TN and very small TP. Therefore, the use of balanced datasets is more preferable. Supplementary File 2: List of the 1302 cancer proteins for Case Study 1. List of the 1302 cancer proteins extracted from the OMIM and HLungDB data-bases, that are not appear in our original training dataset. The 1302 cancer pro-teins are used as an independent test dataset for Case Study 1. Supplementary File 3: List of the 565 potential cancer genes derived from Case Study 2. List of the 565 potential cancer genes derived from four sets of lung cancer mi-croarray data by our method. Five classifiers, including LMT, SimpleCart, J48, LWL and Ridor algorithms were selected for evaluating potential cancer genes under strictly uniformed voting; that is, only the one with five votes which all five classifiers predict as a cancer protein was considered. The 565 potential cancer genes are good targets for future experimental investigation. [file 312047.f1.zip › 312047.f1/paper-cancer-PPI-suppl-files/Figure 1.jpg]

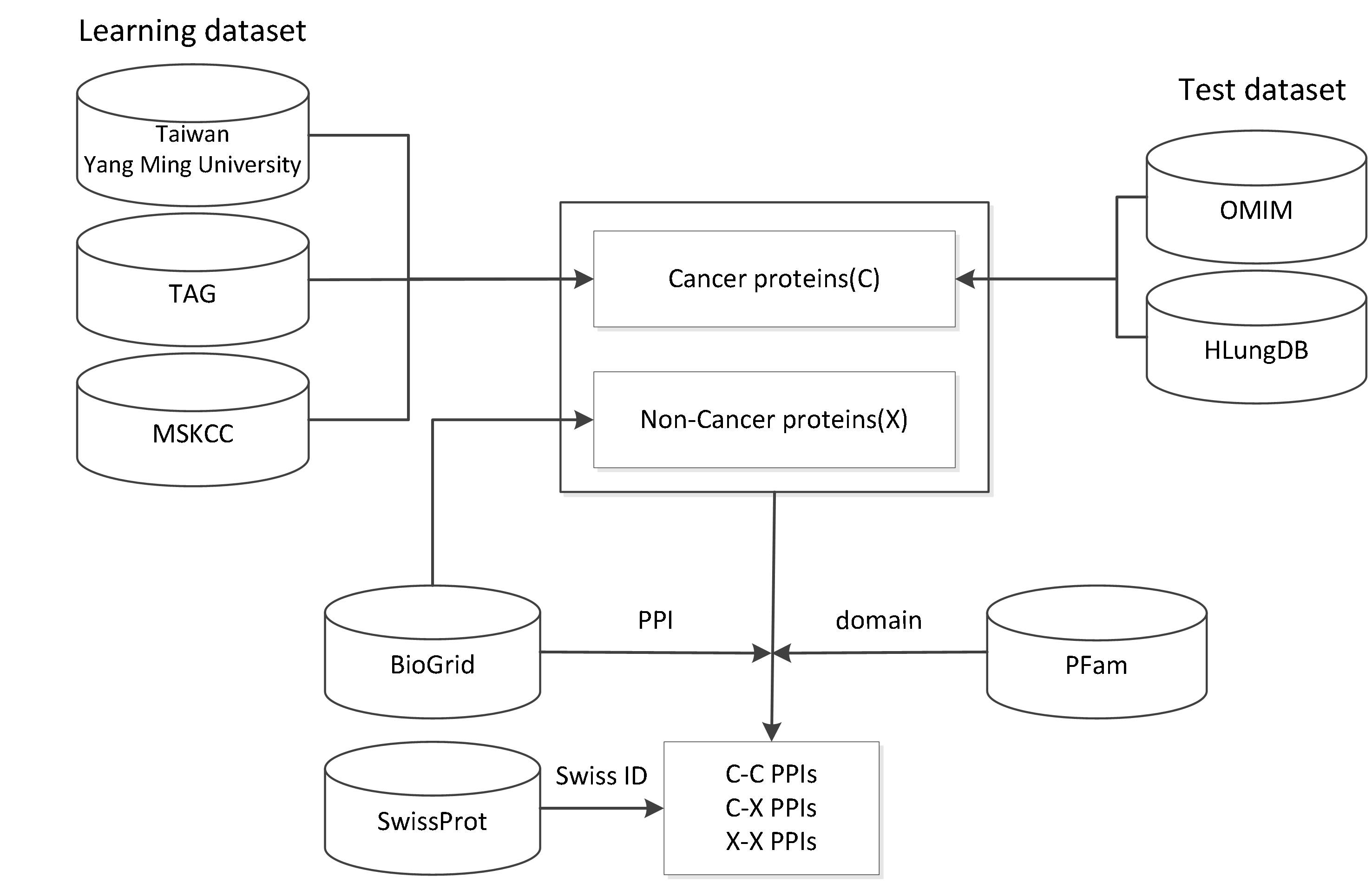

Supplement: Supplementary file 1 — Experimental results with unbalanced data. Here, the corresponding experimental results of the unbalanced dataset are listed in Appendix Tables S1 to S5, where the performance of MCC and PPV is much worse due to the very large TN and very small TP. Therefore, the use of balanced datasets is more preferable. Supplementary File 2: List of the 1302 cancer proteins for Case Study 1. List of the 1302 cancer proteins extracted from the OMIM and HLungDB data-bases, that are not appear in our original training dataset. The 1302 cancer pro-teins are used as an independent test dataset for Case Study 1. Supplementary File 3: List of the 565 potential cancer genes derived from Case Study 2. List of the 565 potential cancer genes derived from four sets of lung cancer mi-croarray data by our method. Five classifiers, including LMT, SimpleCart, J48, LWL and Ridor algorithms were selected for evaluating potential cancer genes under strictly uniformed voting; that is, only the one with five votes which all five classifiers predict as a cancer protein was considered. The 565 potential cancer genes are good targets for future experimental investigation. [file 312047.f1.zip › 312047.f1/paper-cancer-PPI-suppl-files/Figure 2.jpg]

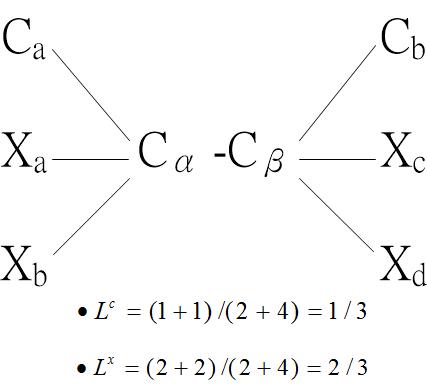

Supplement: Supplementary file 1 — Experimental results with unbalanced data. Here, the corresponding experimental results of the unbalanced dataset are listed in Appendix Tables S1 to S5, where the performance of MCC and PPV is much worse due to the very large TN and very small TP. Therefore, the use of balanced datasets is more preferable. Supplementary File 2: List of the 1302 cancer proteins for Case Study 1. List of the 1302 cancer proteins extracted from the OMIM and HLungDB data-bases, that are not appear in our original training dataset. The 1302 cancer pro-teins are used as an independent test dataset for Case Study 1. Supplementary File 3: List of the 565 potential cancer genes derived from Case Study 2. List of the 565 potential cancer genes derived from four sets of lung cancer mi-croarray data by our method. Five classifiers, including LMT, SimpleCart, J48, LWL and Ridor algorithms were selected for evaluating potential cancer genes under strictly uniformed voting; that is, only the one with five votes which all five classifiers predict as a cancer protein was considered. The 565 potential cancer genes are good targets for future experimental investigation. [file 312047.f1.zip › 312047.f1/paper-cancer-PPI-suppl-files/Figure 3.jpg]

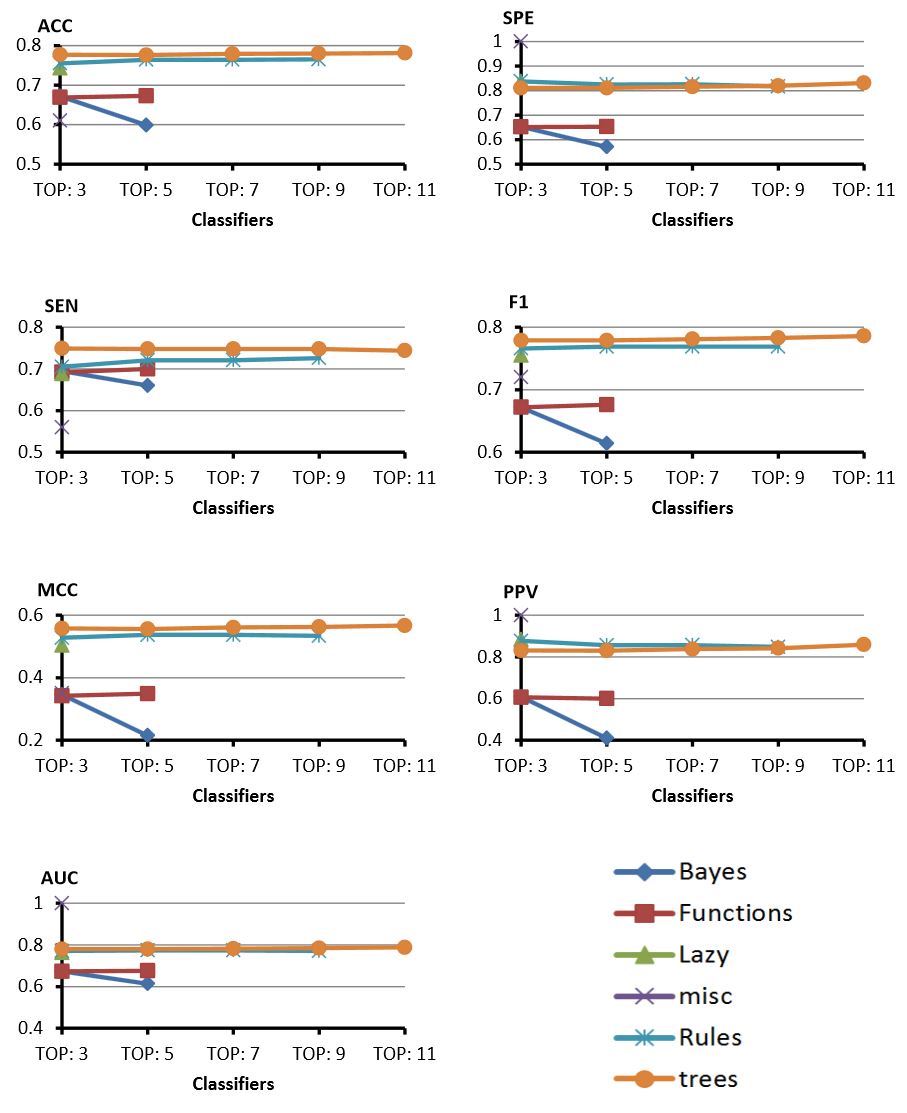

Supplement: Supplementary file 1 — Experimental results with unbalanced data. Here, the corresponding experimental results of the unbalanced dataset are listed in Appendix Tables S1 to S5, where the performance of MCC and PPV is much worse due to the very large TN and very small TP. Therefore, the use of balanced datasets is more preferable. Supplementary File 2: List of the 1302 cancer proteins for Case Study 1. List of the 1302 cancer proteins extracted from the OMIM and HLungDB data-bases, that are not appear in our original training dataset. The 1302 cancer pro-teins are used as an independent test dataset for Case Study 1. Supplementary File 3: List of the 565 potential cancer genes derived from Case Study 2. List of the 565 potential cancer genes derived from four sets of lung cancer mi-croarray data by our method. Five classifiers, including LMT, SimpleCart, J48, LWL and Ridor algorithms were selected for evaluating potential cancer genes under strictly uniformed voting; that is, only the one with five votes which all five classifiers predict as a cancer protein was considered. The 565 potential cancer genes are good targets for future experimental investigation. [file 312047.f1.zip › 312047.f1/paper-cancer-PPI-suppl-files/Figure 4.JPG]

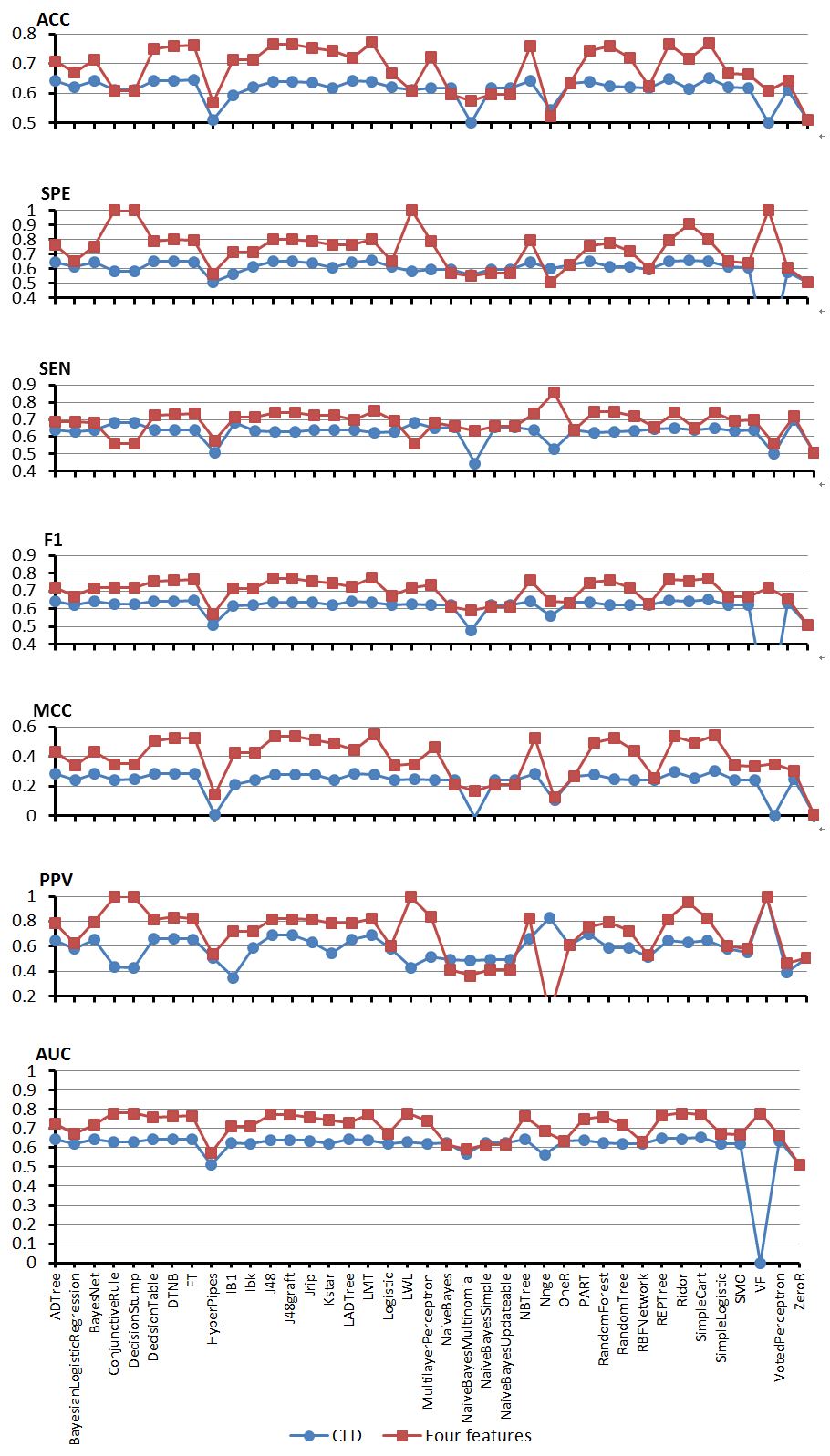

Supplement: Supplementary file 1 — Experimental results with unbalanced data. Here, the corresponding experimental results of the unbalanced dataset are listed in Appendix Tables S1 to S5, where the performance of MCC and PPV is much worse due to the very large TN and very small TP. Therefore, the use of balanced datasets is more preferable. Supplementary File 2: List of the 1302 cancer proteins for Case Study 1. List of the 1302 cancer proteins extracted from the OMIM and HLungDB data-bases, that are not appear in our original training dataset. The 1302 cancer pro-teins are used as an independent test dataset for Case Study 1. Supplementary File 3: List of the 565 potential cancer genes derived from Case Study 2. List of the 565 potential cancer genes derived from four sets of lung cancer mi-croarray data by our method. Five classifiers, including LMT, SimpleCart, J48, LWL and Ridor algorithms were selected for evaluating potential cancer genes under strictly uniformed voting; that is, only the one with five votes which all five classifiers predict as a cancer protein was considered. The 565 potential cancer genes are good targets for future experimental investigation. [file 312047.f1.zip › 312047.f1/paper-cancer-PPI-suppl-files/Figure 5.JPG]

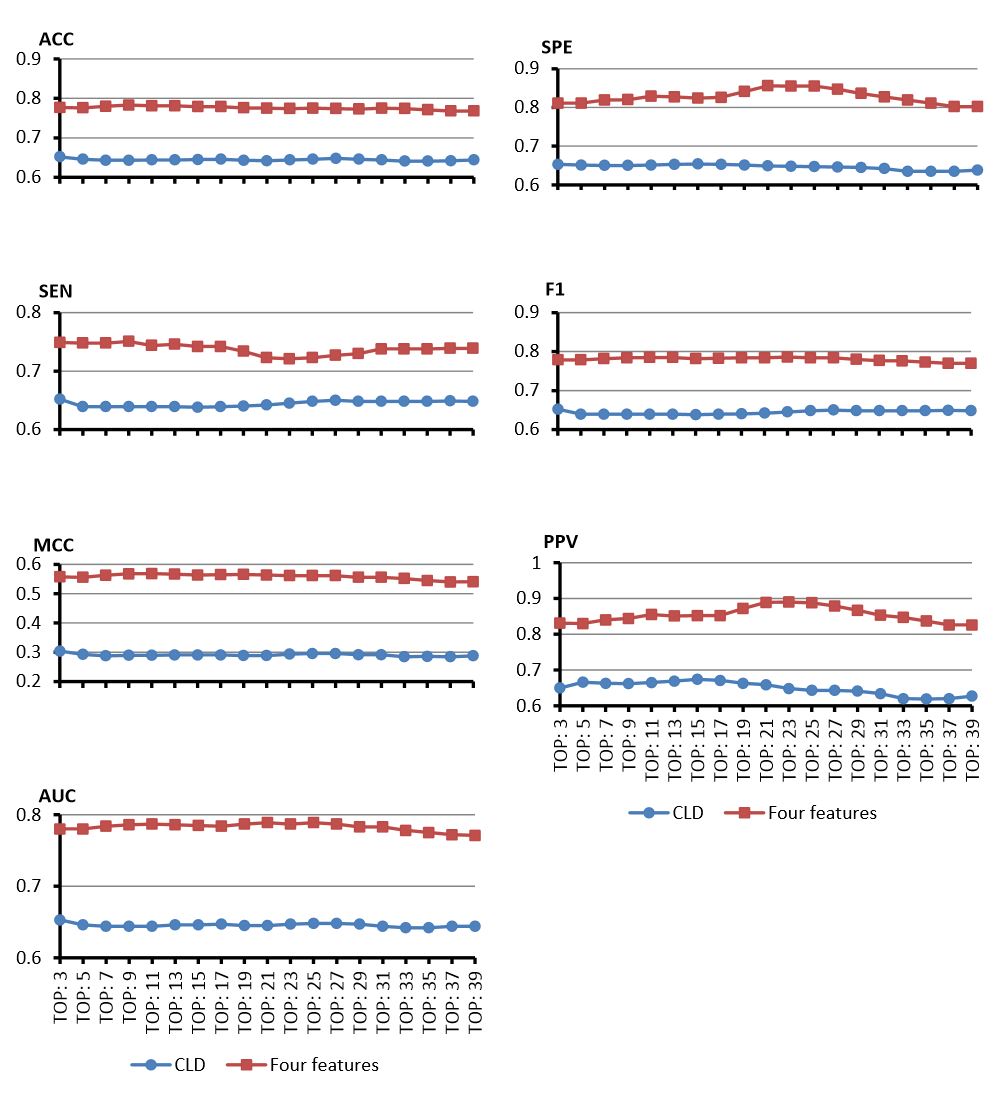

Supplement: Supplementary file 1 — Experimental results with unbalanced data. Here, the corresponding experimental results of the unbalanced dataset are listed in Appendix Tables S1 to S5, where the performance of MCC and PPV is much worse due to the very large TN and very small TP. Therefore, the use of balanced datasets is more preferable. Supplementary File 2: List of the 1302 cancer proteins for Case Study 1. List of the 1302 cancer proteins extracted from the OMIM and HLungDB data-bases, that are not appear in our original training dataset. The 1302 cancer pro-teins are used as an independent test dataset for Case Study 1. Supplementary File 3: List of the 565 potential cancer genes derived from Case Study 2. List of the 565 potential cancer genes derived from four sets of lung cancer mi-croarray data by our method. Five classifiers, including LMT, SimpleCart, J48, LWL and Ridor algorithms were selected for evaluating potential cancer genes under strictly uniformed voting; that is, only the one with five votes which all five classifiers predict as a cancer protein was considered. The 565 potential cancer genes are good targets for future experimental investigation. [file 312047.f1.zip › 312047.f1/paper-cancer-PPI-suppl-files/Figure 6.JPG]
